# Supplementary material for: The off-hour effect on mortality in traumatic brain injury according to age group
Source: PLoS One. 2023 Mar 16;18(3):e0282953. doi: 10.1371/journal.pone.0282953 (PMC10019691; doi:10.1371/journal.pone.0282953)
Supplement: Attachment — Submitted filename: revisionletter.docx [file pone.0282953.s001.docx]

PONE-D-22-13485

The Off-Hour Effect on Mortality in Traumatic Brain Injury According to Age Group

Journal: PLOS ONE

Editor comments

(1) Please ensure your names are spelled correctly throughout the manuscript (Ro is mentioned in Author Contributions, which I hope is a typo)

ANSWER: Thank you for review. We revised ‘Author Contributions’.

(2) Funding Acknowledgement: Please indicate whether the grant had an influence or otherwise restricted the research.

ANSWER: Thank you for review. We added funding statement within our cover letter.

(3) Could you provide I little more information on EMT level-1 and level-2 differences in competencies?

ANSWER: Thank you for review. We added some explanation the difference between level-1 and level-2 EMTs.

(REVISION: METHODS-*Study design, setting, and population*) The level-1 EMTs can be performed with high grade procedures including advanced airway and intravenous line access compared to level-2 EMTs

(4) We note that you have provided funding information that is not currently declared in your Funding Statement. However, funding information should not appear in the Acknowledgments section or other areas of your manuscript. We will only publish funding information present in the Funding Statement section of the online submission form. 
Please remove any funding-related text from the manuscript and let us know how you would like to update your Funding Statement. Currently, your Funding Statement reads as follows:

"The funders had no role in study design, data collection and analysis, decision to publish, or preparation of the manuscript"

Please include your amended statements within your cover letter; we will change the online submission form on your behalf.

ANSWER: Thank you for review. We remove funding-related text from the manuscript, update funding statement section, and added funding statement within our cover letter.

Reviewers' comments:

Reviewer #1: In a single center population of TBI patients between 2017-2020, the authors found no significant difference in mortality in patients visiting ED during off hours. The effect measure of ED admission during off hours on mortality was significant in patients aged 0-17 years compared to other age groups. Additionally, 85% of TBI patients presented during off hours and there were no differences in the proportion of surgical intervention between on- and off- hours.

Abstract:
- Would not state TBI is a surgical emergency, as 80% are mild and even moderate to severe may not always require surgery.

Answer: Thank you for the review. We edited the sentence according to your advice.

<Revision: Abstract-*Background*>

Traumatic brain injury (TBI) is a time-sensitive and life-threatening medical condition.

- Please include N. Please include how TBI was defined at the study institution. Please include more relevant data in the results section.

Answer: Thank you for the review. We added the sentence according to your advice.

<Revision: Abstract-*Results*>

: A total of 2086 patients with TBI with intracranial injury who were transported by EMS were enrolled in our registry.

: In the interaction analysis, the effect measure of ED admission during off-hours on mortality was significant among younger people (0—17 years: 1.17 [1.04—1.30]), compared to that in other age groups (18—64 years: 1.04 [0.46—2.37] and 65—100 years (0.97 [0.41—2.27])).

- I'm not sure the conclusion supports that any differences are currently present between daytime and nighttime emergency care.
Answer: Thank you for the review. Although a causal relationship has not been proven that there is a difference between emergency care and mortality when young TBI patients visit off-hours, based on previous studies showing that off-hour visit increased mortality and decreased surgical intervention, our study tried to prove that difference in mortality among off-hor visit differs according to age.

Introduction:
- Would not state TBI is a surgical emergency, similar to above.

Answer: Thank you for the review. We edited the sentence according to your advice.

<Revision: Introduction>

TBI is a time-sensitive and life-threatening medical condition

Methods:
- Authors need to define what constitutes "TBI" at their site. This alone could be a limitation depending on how it is defined.

Answer: Thank you for the review.

<Revision: Methods-*Study desing, setting, and population*>

The inclusion criteria are patients with TBI over 18 years of age who visited trauma center using emergency medical services (EMS) within 72 hours of injury and had intracranial hemorrhage and/or diffuse axonal confirmed by a radiological examination.

- Per the authors, "The main outcome measure was mortality at hospital discharge. The secondary outcome was surgical intervention within 6 hours of ED admission. The main exposure variable was arrival time at ED admission (off-hour vs. working-hour). We defined daytime as 09:00 AM to 17:59 PM and nighttime as 18:00 PM to 08:59 AM; weekdays as Monday to Friday; and weekends as Saturday, Sunday, and national holidays, based on a previous study." As such, there should be a limitation discussed as 6 pm and 9 am are hardly accepted as "nighttime" at most institutions.

Answer: Thank you for the review. We discussed in Limitation section according to your advice.

<Revision: Discussion>

The inclusion criteria are patients with TBI over 18 years of age who visited trauma center using emergency medical services (EMS) within 72 hours of injury and had intracranial hemorrhage and/or diffuse axonal confirmed by a radiological examination.

- Unclear why the authors controlled for so few variables while using a multivariable regression despite evaluating much larger initial cohorts. We know variables such as psychiatric history, history of prior TBIs, etc, can lead to poorer outcome.

Answer: Thank you for the review. We clearly defined the exclusion criteria for the study population according to your advice.

<Revision: Methods-*Study desing, setting, and population*>

Patients with neurological disorders, psychiatric disorders, and terminal cancer, pregnant women, patients with penetrating brain injury, and patients transferred to the participating hospital ED after surgery at another hospital were also excluded from the study.

- The Pediatric cohort is usually a completely separate population in characteristics and (in part) response to TBI, however were included as part of the main analysis in this study.
Answer: Thank you for the review. It is very important to point out that the incidence and progression patterns of pediatric TBI patients are somewhat different from those of adults. However, in our study, rather that looking at the difference in clinical outcomes of TBI between each age group, we look at the difference in clinical outcomes according to the time of ED visit in each age group.

Limitations:
- Need to be augmented per comments above.
Answer: Thank you for the review. We added some sentences about limitations of our study according to your advice.

Discussion:
- The statement "EDs should, thus, be designed such that patients can expect the same quality of emergency care regardless of admission time." should be tempered/revised, as there is no recent evidence that ER care changes based on the hour.

Answer: Thank you for the review. We revised sentence according to your advice.

<Revision: Discussion>

it is necessary to pay more attention to that there is no difference in the quality of treatment regardless of the day and time of admission.

- How can the results of this study change and/or improve practice?

Answer: Thank you for the review. As described in the Discussion section of our study, the results of our study do not causally explain the decline in a quality of care for pediatric TBI patients at off-hours, but, it is theoretical basis for the need for pay more attention to that there is no difference in the quality of treatment regardless of the day and time of admission for the TBI patients, especially pediatric patients.

Reviewer #2:

1. term seniors is. not universal (my pdf page 11)

Answer: Thank you for the review. We corrects the word according to your advice

<Revision: Introduction>

more experienced doctor

2. weekdays and holidays are combined...are there Monday holidays like the US ?

Answer: Thank you for the review. As you pointed out, the definition of weekday is clarified.

<Revision: Methods-*Main outcome and variables*>

weekdays as Monday to Friday except national holidays

3. am I correct these patients have isolated head injury. if they have a wrist fracture that only required splint, or a isolated ribfracture, were they excluded ?

Answer: Thank you for the review. As discussed in the Method section, patients who had TBI accompanied by injuried at other sites were excluded.

<Revision: Methods-Study design, setting, and population>

Patients who had TBI accompanied by injuries at other sites, those with no information of hospital admission, those who underwent surgical intervention, and those who died were excluded.

4. is time from ED presentation to OR different in the weekday vs weekend group. are neurosurgeons available in the hospital during the day and night ? do they need to travel during these times from another office or home. I assume the OR staff/OR are 24/7/365.

Answer: Thank you for the review. In principle, the level-1 trauma center has specialists such as neurosurgeon on standby 24/7/365, and personnel for surgical intervention are also on standby at the hospital. However, although not investigated in our study, we assumed that the number of personnel for surgical intervention and the activation process will not be smoother in off-hours.

5. maybe the important variable

Answer: Thank you for the review. But we did not understand the meaning of your point. If possible, please explain again.

6. table 1 ..what does intentionality mean

Answer: Thank you for the review. We added sentence about intentionality.

<Revision: Methods-*Main outcome and variables*>

intentionality (including self inflicted and interpersonal acts of violence intended to cause injury)

7. the p value ...what comparisons do they represent ...so age ...is it the 3 categories 0-17, 18-64 and ≥65 vs day and night? are these multiple comparisons still significant after a multiple comparison (Bonferroni) correction? same is true for all these other 3x2 , 5x2 ,4x2 comparisons

Answer: Thank you for the review. The p-values in Table 1 and 2 show the significance of the difference between each group. The p-values of the interaction analysis showe the significance of the odds ratios of the off-hour for each age when the working hour is used as a reference in each study outcomes.

8. seems like there is overlaps in table 1 and 2....there is day vs night, weekday vs weekend, then in table 2 its really the same but now defined as off hours vs working hours. Why have the two and not just simply with on hours and off hours ?

Answer: Thank you for the review. Table 1 shows the characteristics of patients according to time of day and day of week, and Table 2 presents the differences in patient characteristics by dividing them into off-hour and working-hour. These definitions are clearly written in Method section.

<Revision: Methods-*Main outcome and variables*>

We defined off-hour when it was nighttime or weekends, and defined other times as working-hour.

9. it looks like these multiple comparisons also exist in other tables
so for example table 4 , mortality at hospital dc is p < .05. for a 3x2 table , 6 comparisons the level of significance is not .05 , but actually .05 /6 , significance adjusted 0.01250.
so your report of significance if not corrected at < .05 actually is. not significant between .01250 and .05.

Answer: Thank you for the review. We revised Table 4 according to your advice.

(Revision: Table 4)

| Whole TBI patients | Arrival time | Mortality at hospital discharge | Surgical intervention |
| --- | --- | --- | --- |
| Age |  |  |  |
| 0-17 |  |  |  |
|  | Working-hours | reference | reference |
|  | Off-hours | 1.17 (1.04-1.30) | 1.52 (0.58-3.95) |
| 18-64 |  |  |  |
|  | Working-hours | reference | reference |
|  | Off-hours | 1.04 (0.46-2.37) | 1.04 (0.70-1.55) |
| 65- |  |  |  |
|  | Working-hours | reference | reference |
|  | Off-hours | 0.97 (0.41-2.27) | 0.87 (0.56-1.35) |
| Subdural hemorrhage patients |  |  |  |
| Age |  |  |  |
| 0-17 |  |  |  |
|  | Working-hours | reference | reference |
|  | Off-hours | 1.14 (0.97-1.33) | 1.47 (0.57-3.81) |
| 18-64 |  |  |  |
|  | Working-hours | reference | reference |
|  | Off-hours | 0.96 (0.43-2.17) | 0.88 (0.60-1.30) |
| 65- |  |  |  |
|  | Working-hours | reference | reference |
|  | Off-hours | 1.09 (0.47-2.51) | 0.87 (0.56-1.34) |
