# Supplementary material for: The off-hour effect on mortality in traumatic brain injury according to age group
Source: PLoS One. 2023 Mar 16;18(3):e0282953. doi: 10.1371/journal.pone.0282953 (PMC10019691; doi:10.1371/journal.pone.0282953)
Supplement: Attachment — Submitted filename: ResponseToReviewers_3rd.docx [file pone.0282953.s002.docx]

**Author’s reply to reviewers' comments:**

On behalf of authors, thank you for the very valuable comments by the reviewer on our paper. We have attempted to address every point commented on by the reviewer in the revised manuscript. While we believe that we have addressed all of the reviewer’s concerns, we would be more than pleased to write additional revisions if needed.

We highlighted all changes in red. Author’s answers or explanations are in blue.

Thank you for your positive response to my request for expedited publication.

Correspondent author

Hyun Ho Ryu, MD, PhD

PONE-D-22-13485

The Off-Hour Effect on Mortality in Traumatic Brain Injury According to Age Group

Journal: PLOS ONE

Reviewer #4:

(1) Retrospective single center review examining outcomes for TBI patients by time off day and day of week of presentation. The authors demonstrate that outcomes are similar for all patients regardless of when they present. There is a signal for patients 0-17 where mortality seems to be increased at off-hours time, although this is based on only 3 deaths - and may statistically significant but clinically less so.
ANSWER: Thank you for the review. We agree with the fact that the result of our study that the mortality at 0—17-year-old increases in statistically significant, but clinically has limitations, and we explained the limitation section.

(REVISION: Discussion): Second, in our registry, there were only 3 deaths in 0—17 years, although statistically significant, caution is needed in clinical confirmation.

(2) In general, outcomes are dependent on anatomic injury, presenting physiology, and comorbidities. Do the authors have any data on comorbidities in their study population (especially the older patients).
ANSWER: Thank you for the review. We added variables of comorbidities including hypertension and diabetes mellitus in multivariable logistic regression analysis according to your advice.

(REVISION: Table 1,2,3)

(3) For the 0-17 age group, the findings are of interest, but the authors need to better describe specifically who cares for this patient population during the day as well as during off hours. Do you use pediatric specialists (and especially pediatric neurosurgeons and / or neurointensivists) and are any specialists available 24/ 7 or only during the workdays?
ANSWER: Thank you for the review. Our trauma center does not have a specialist in charge of only pediatrics, and cares by department regardless of age. I add these to the method section.

(REVIOSN: Methods-*Study design, setting, and population*): These protocols are the same regardless of age, and there is no medical staff dedicated to a specific age group.

(4) Finally, the conclusions should include the equivalent outcomes for the vast majority of patients. This speaks well of the trauma center / system that you have set up and should not be minimized.

ANSWER: Thank you for the review. As you commented, the conclusions of our study are limited to generalization because they are the results of the EMS and ED environments in Korea. We deleted this sentence (‘EDs should, thus, be designed such that patients can expect the same quality of emergency care regardless of admission time.’) according to your advice.
